# iNOS-Producing Inflammatory Dendritic Cells Constitute the Major Infected Cell Type during the Chronic *Leishmania major* Infection Phase of C57BL/6 Resistant Mice

**DOI:** 10.1371/journal.ppat.1000494

**Published:** 2009-06-26

**Authors:** Carl De Trez, Stefan Magez, Shizuo Akira, Bernhard Ryffel, Yves Carlier, Eric Muraille

**Affiliations:** 1 Laboratoire de Parasitologie, Faculté de Médecine, Université Libre de Bruxelles, Brussels, Belgium; 2 Department of Molecular and Cellular Interactions, Vlaams Interuniversitair Instituut voor Biotechnologie, Vrije Universiteit Brussel, Brussels, Belgium; 3 Department of Host Defense, Research Institute for Microbial Diseases, Osaka University Yamadaoka, Suita City, Osaka, Japan; 4 University of Orleans, Transgenose Institute, CNRS, UMR 6218, Orleans, France; University of Wisconsin-Madison, United States of America

## Abstract

*Leishmania major* parasites reside and multiply in late endosomal compartments of host phagocytic cells. Immune control of *Leishmania* growth absolutely requires expression of inducible Nitric Oxide Synthase (iNOS/NOS2) and subsequent production of NO. Here, we show that CD11b^+^ CD11c^+^ Ly-6C^+^ MHC-II^+^ cells are the main iNOS-producing cells in the footpad lesion and in the draining lymph node of *Leishmania major*-infected C57BL/6 mice. These cells are phenotypically similar to iNOS-producing inflammatory DC (iNOS-DC) observed in the mouse models of *Listeria monocytogenes* and *Brucella melitensis* infection. The use of DsRed-expressing parasites demonstrated that these iNOS-producing cells are the major infected population in the lesions and the draining lymph nodes. Analysis of various genetically deficient mouse strains revealed the requirement of CCR2 expression for the recruitment of iNOS-DC in the draining lymph nodes, whereas their activation is strongly dependent on CD40, IL-12, IFN-γ and MyD88 molecules with a partial contribution of TNF-α and TLR9. In contrast, STAT-6 deficiency enhanced iNOS-DC recruitment and activation in susceptible BALB/c mice, demonstrating a key role for IL-4 and IL-13 as negative regulators. Taken together, our results suggest that iNOS-DC represent a major class of Th1-regulated effector cell population and constitute the most frequent infected cell type during chronic *Leishmania major* infection phase of C57BL/6 resistant mice.

## Author Summary

*Leishmania* spp. are protozoan parasites infecting a variety of mammals, including humans and mice. Much information has been gleaned from murine models of *Leishmania major* infection. The control of *L. major* infection by resistant C57BL/6 mice requires the secretion of type 1 (Th1) cytokines (i.e. IFN-γ) by T cells as well as the expression of inducible nitric oxide synthase (iNOS) by phagocytic cells. Conversely, susceptible BALB/c mice are unable to control infection and develop a type 2 (Th2) immune response characterized by the secretion of IL-4 and IL-13 cytokines. In this study, we showed that the main iNOS-producing cells in the lesion and the draining lymph node are phenotypically similar to iNOS-producing “inflammatory” dendritic cells (DC), which are already described in the mouse models of *Listeria monocytogenes* and *Brucella melitensis* infection. Our data also highlighted a strong association between the recruitment and activation of these inflammatory DC and the resistance to *L. major* infection. In addition, we showed that iNOS production by these inflammatory DC is positively regulated by Th1 response and negatively by Th2 response. Taken together, our results provide new insights into how innate and adaptive immune responses fight *L. major* infection. A better understanding of the mechanisms regulating inflammatory DC recruitment and activation could lead to new therapeutic strategies against *Leishmania* infection.

## Introduction

*Leishmania* spp. are protozoan parasites belonging to the *Trypanosomatidae* family. They are transmitted by phlebotomine sand flies to a variety of mammals, including humans and mice (reviewed in references [1],[2],[3]). These organisms, under amastigote form, reside and multiply in late endosomal compartments of host phagocytic cells. Clinical manifestations of *Leishmania* infection vary with regards to the particular parasite species, the host immune response, and genetic factors, and much information has been gleaned from murine models of *Leishmania major* infection. The control of *L. major* and the development of long-lasting resistance require the interleukin (IL)-12 dependent differentiations of type 1 CD4^+^ T helper cells (Th1). The secretion of interferon (IFN)-γ by Th1 cells induces the expression of inducible nitric oxide synthase (iNOS, also termed NOS2) by phagocytic cells, leading to the production of nitric oxide (NO) [4]. iNOS expression remains high in chronically infected, but clinically healthy mice, and is absolutely crucial for the sustained control of *L. major* [5],[6],[7]. Genetically resistant mouse strains (e.g. C57BL/6) develop a strong Th1 response and restrict the spread of local parasite infection. In contrast, non-healing mouse strains (e.g. BALB/c) mount a Th2 response associated with high level IL-4 and IL-13 production by CD4^+^ T cells. C57BL/6 mice lacking MyD88 [8], CD40 [9], IL-12 [10], IFN-γ [11] or CCR2 [12] display a Th2-skewed response, associated with a severe reduction in iNOS expression and high tissue parasite burdens. In turn, BALB/c mice lacking IL-4 [13], IL-13 [14] or STAT-6 [15] develop a Th1-skewed response and are resistant to *Leishmania* infection.

Dendritic cells (DC) play an essential role in initiating and shaping Th1 protective responses in *Leishmania* infection, mostly through production of IL-12p70 [16],[17],[18]. In the last decade, it has become clear that DC represent a highly heterogeneous cell population, with various subsets being defined by their differential expression of various cell surface markers and specialized functions [19],[20],[21]. Recently, a population of DC expressing CD11b^+^ CD11c^+^ LY-6C^+^ MHC-II^+^ and high levels of iNOS protein (termed inflammatory DC or TNF-iNOS-producing DC (Tip-DC)) [22],[23] has been implicated in the resistance to infection by intracellular bacteria (e.g. *Listeria monocytogenes* [22] and *Brucella melitensis*) [24]. These observations suggest that these cells might be a potential source of iNOS during infection by *Leishmania*.

In the present study, using immunofluorescent microscopy and *ex vivo* flow-cytometric analysis, we demonstrated that inflammatory DCs are the main producers of iNOS *in vivo* during the course of *L. major* infection. The recruitment of inflammatory DC was dependent upon CCR2 expression, and the induction of iNOS expression in these cells required the development of a local Th1 microenvironment, as demonstrated by the reduced frequency of iNOS^+^ inflammatory DC in MyD88^−/−^, CD40^−/−^, IL-12^−/−^ and IFN-γ^−/−^ mice. In contrast, a Th2 environment inhibited the local differentiation of iNOS^+^ inflammatory DC, as an enhanced frequency of iNOS^+^ inflammatory DC was observed in STAT-6^−/−^ BALB/c mice.

## Results

### Recruitment of iNOS-producing inflammatory DC during the course of *L. major* infection

**Figure 1:**
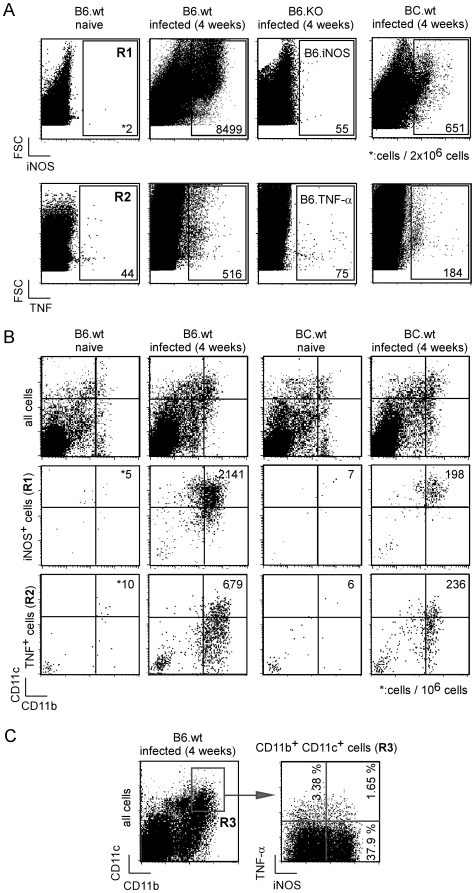
iNOS- and TNF-α-producing cells following *Leishmania* infection. Wild-type, iNOS-and TNF-α -deficient C57BL/6 as well as BALB/c mice (4 per group) were injected into the footpad with PBS or *L. major* promastigotes. Mice were sacrificed four weeks post-infection and the draining popliteal lymph nodes were collected and analyzed by flow cytometry. Cells were priorly gated according to size and scatter to exclude dead cells and debris from analysis. *A*, Total pooled lymph node cells were analyzed for Forward Size Scatter (FSC), iNOS and TNF-α expression. *B–C*, Total pooled lymph node cells as well as iNOS^+^ cells (R1 gate, Figure 1A) and TNF^+^ cells (R2 gate, Figure 1A) were analyzed for CD11b and CD11c expression. *C*, CD11b^hi^ CD11c^hi^-gated cells (R3 gate) were analyzed for iNOS and TNF-α expression. Numbers in Figure 1A and 1B indicate the number of positive cells per 2×10^6^ and 10^6^ cells acquired from total lymph node cells, respectively. Numbers in Figure 1C indicate the percentage of cells expressing iNOS and/or TNF-α in the CD11b^hi^ CD11c^hi^-gated population (R3 gate). Data are representative of 4 independent experiments.

Monitoring of *L. major*-induced lesion size in wild-type C57BL/6 (B6.WT), B6.iNOS, B6.TNF-α mice and wild-type BALB/C (BC.WT) mice confirmed the previous results of our group and others, showing an important contribution of both iNOS enzymatic activity [5],[6],[7] and TNF-α [25] in the resistance of B6.WT mice to *L. major* infection (Figure S1.A). However, the severity of the lesions in the footpads four weeks post-infection (p.i.) highlighted a more crucial role for iNOS compared to TNF-α in this pathogenic model (Figure S1.A and S1.B). To this point, the exact nature of the cell type(s) responsible for the production of iNOS and TNF-α *in vivo* has remained unclear, and we therefore attempted to directly identify them in the draining lymph node (LN) four weeks after primary *L. major* infection (Figure 1A, gate R1 and R2, respectively). Approximately 8500 cells per 2×10^6^ (0.4%) of the cells analyzed appeared positive for iNOS after intracellular staining, whereas 20-fold fewer cells expressed detectable level of TNF-α. A time course analyses of iNOS and TNF-α expression by LN cells from B6.WT and BC.WT infected mice demonstrated that four weeks p.i. was the peak expression for these two proteins (data not shown).

Next, the phenotype of the iNOS^+^ and TNF-α^+^ LN cells in resistant B6.WT and susceptible BC.WT mice was examined. The majority of iNOS^+^ and TNF-α^+^ cells expressed high levels of CD11b and CD11c, characteristic of inflammatory DC (Figure 1B). Among the iNOS^+^ (R1 gate) and TNF-α^+^ (R2 gate) LN cells, resistant B6.WT mice displayed ∼10-fold and ∼3-fold more CD11b^hi^ CD11c^hi^ cells compared to susceptible BC.WT mice, respectively (Figure 1B). The analysis of TNF-α and iNOS expression in the CD11b^hi^ CD11c^hi^ cells-gated population of infected B6.WT resistant mice demonstrated that a high frequency (37.9%) of these cells expressed iNOS, whereas only a negligible fraction expressed either TNF-α (3.3%) or both iNOS and TNF-α (1.6%) (Figure 1C, gate R3). Together, these results demonstrated that iNOS-producing CD11c^hi^ CD11b^hi^ DC are recruited to the draining LN to a much higher degree in infected B6.WT mice compared to infected BC.WT mice, and that iNOS expression by these cells was much more prominent than TNF-α expression.

### iNOS-producing cells during *Leishmania major* infection phenotypically resemble to inflammatory DC

**Figure 2:**
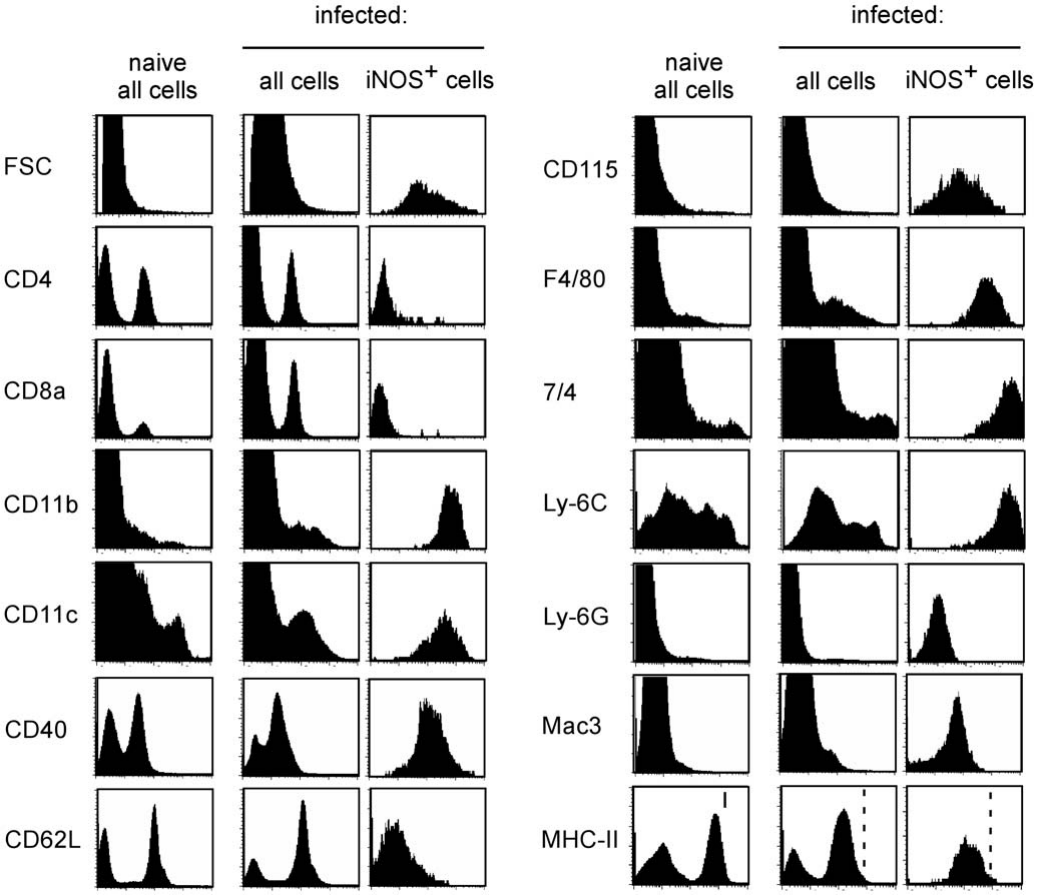
Phenotype of iNOS-producing cells following *Leishmania* infection. C57BL/6 mice were injected into the footpad with PBS or *L. major* promastigotes. Mice were sacrificed four weeks post-infection and the draining popliteal lymph nodes were collected. Total pooled lymph node cells from uninfected and infected mice as well as iNOS^+^-gated cells from infected mice were analyzed by flow cytometry for FSC, CD4, CD8α, CD11b, CD11c, CD40, CD62L, CD115, F4/80, 7/4, Ly-6C, Ly-6G, Mac3 and MHC-II expression. Results are derived from at least *n* = 5 mice and the data are representative of 3 independent experiments.

To further characterize iNOS^+^ cells in the draining LN of *L. major* infected B6.WT mice, an extensive phenotypic characterization of these cells was performed by flow cytometry (Figure 2). iNOS-producing cells also expressed F4/80, CD115, 7/4, Ly-6C and Mac3 markers, but did not stain for CD4, CD8α and Ly-6G markers. Additionally, CD40 and MHC-II molecules were also highly expressed, suggesting a potential antigen presenting function of these cells. Thus, the iNOS-producing cells induced during *L. major* infection phenocopied the surface phenotype of the “TNF-α/iNOS-producing DC, TipDC” or “inflammatory DC”, described in other infectious [22],[24] and non infectious models [26].

### *In situ* characterization of iNOS-producing cells in the draining lymph node

To gain further insight into the infection level of iNOS-expressing DC *in vivo*, we infected mice with a strain of *L. major* which stably expresses the Discosoma Red (DsRed) fluorescent tracer protein (DsRed-Leish) [27]. As reported by the group of C. Ardavin [18], flow cytometric characterization of infected cells in draining lymph nodes showed CD11b^+^ DsRed-Leish^+^ cells and CD11c^+^ DsRed-Leish^+^ cells (Figure S2.A). However, we also observed the unexpected presence of IgD^+^ DsRed-Leish^+^ cells and CD3ε^+^ DsRed-Leish^+^ cells (Figure S2.A). As this approach did not exclude the possibility that during purification (i) non infected phagocytic cells might become infected *in vitro* or (ii) extracellular parasite adhere to cells, we performed an experiment to detect “false-positive” DsRed-Leish^+^ cells (Figure S2.B). When LN cells from naïve uninfected BALB/C mice expressing the T cell congenic marker CD90.1 are mixed during *ex vivo* purification with LN cells from DsRed-Leish-infected BALB/C expressing the congenic marker CD90.2, we found DsRed-Leish^+^ LN CD90.1^+^ cells. This result clearly demonstrated that the manipulation of infected cells/tissues *ex vivo* can result in the apparition of “false-positive” DsRed-Leish^+^ cells that are normally not infected *in situ*. Moreover, when cytospins were performed on sorted DsRed-Leish^+^ cells from infected BC.WT LN, we observed that a fraction of these cells do not exhibit an intracellular localisation of the parasite, but just display it bound to their cell surface (Figure S2.C). Consequently, these very important technical considerations led us to restrict our analysis of the cellular tropism of *L. major* infection in draining LN to *in situ* analysis.

Large DsRed-Leish infected tissue sections were scanned at high resolution with a motorized fluorescent microscope. Resulting images were analyzed with the “Colocalization” module (an AxioVision program, Zeiss) to precisely identify the phenotype of both infected and iNOS-producing cells. These newly developed analysis techniques provide quantitative and statistically significant data, and avoid the potential loss of cell populations that might occur during the harvesting and processing of tissue before *ex vivo* analyses, such as cytofluorometric analysis.

**Figure 3:**
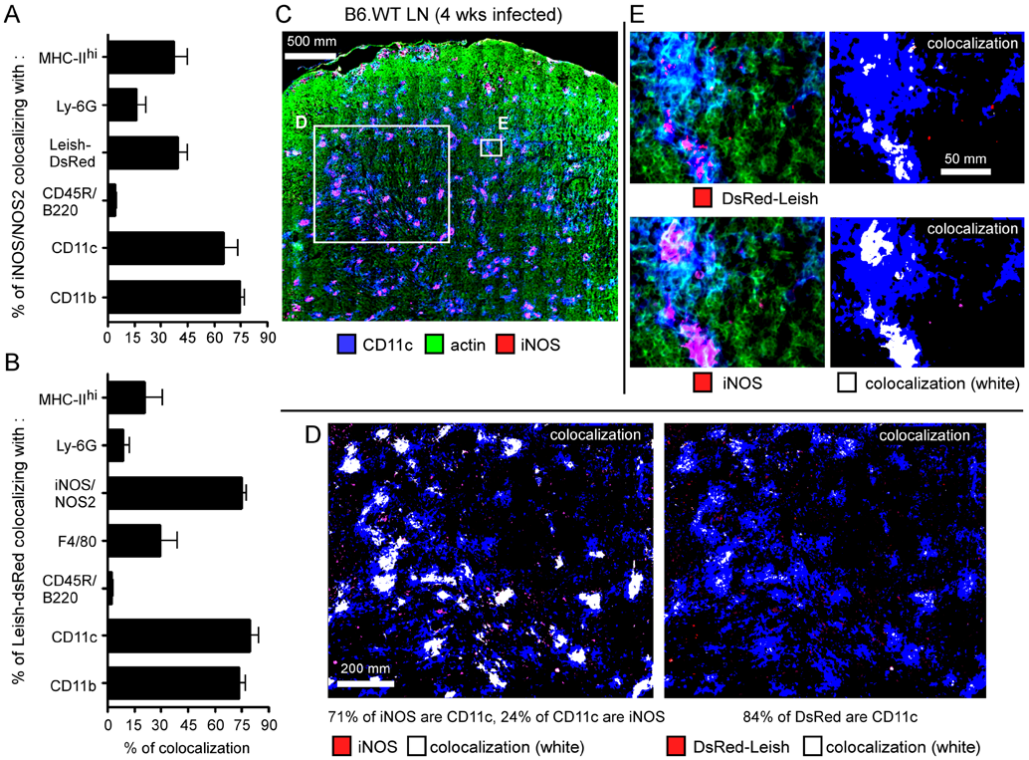
Characterization of iNOS- and DsRed-expressing cells in infected LN. Wild type C57BL/6 mice were injected into the footpad with PBS or DsRed expressing *L. major* promastigotes (DsRed-Leish). Mice were sacrificed four weeks post-infection, the draining popliteal lymph nodes were collected and examined by immunohistofluorescence. *A*, The percentage of iNOS^+^ cells that colocalize with MHC-II^hi^-, Ly-6G-, CD45R/B220-, CD11c-, CD11b-expressing cells and DsRed-Leish. *B*, The percentage of DsRed-Leish that colocalize with MHC-II^hi^-, Ly-6G-, iNOS-, F4/80-, CD45R/B220-, CD11c- and CD11b-expressing cells. The bars are the mean±SD from at least 3 LN sections per LN from 8 mice. *C–E*, Immunofluorescence analysis of CD11c, actin, DsRed and iNOS expression. Panels are color-coded with the text for the antigen or DsRed-Leish examined as well as the colocalization. *D–E*, Higher magnification view of Figure 3C, as indicated. Numbers in Figure 3D indicate the percentage of colocalizing cells in the upper panel. Scale bar = 500, 200 and 50 µm, as indicated. Data are representative of 3 independent experiments.

By performing a colocalization analysis of large scanned LN surfaces, it was confirmed that iNOS expression largely colocalized with CD11b^+^ and CD11c^+^ cells (75% and 65–70% of the iNOS^+^ surface, respectively), with approximately 40% and 35% also exhibiting DsRed signal and high expression levels of MHC-II, respectively (Figure 3A). Using the same analysis technique, ∼75% of the DsRed-Leish^+^ surface colocalized with iNOS^+^, CD11b^+^ and CD11c^+^ cells, but <30% did with Ly-6G and MHC-II^hi^ (Figure 3B). The Figures 3C–E depicted representative examples of colocalization between CD11c^+^, iNOS^+^ and DsRed-Leish^+^ in infected draining LN sections. They also illustrated the aggregated distribution of these cells within the draining LN. CD45R/B220 expression (largely a B-cell marker) displayed very little colocalization with iNOS^+^ and DsRed-Leish^+^ surface (<5%, Figure 3A and 3B), and likely represented the general degree of non-specific colocalization (due to cell superposition in the section) when analyzing tissue sections averaging 10 µm thickness. We did not observe any iNOS staining in B6.iNOS^−/−^ mice (data not shown).

Analyses of tissue sections showed that approximately 70% of iNOS^+^ cell surface expressed CD11b and CD11c markers, yet only ∼35% colocalized with MHC-II^hi+^ surface. This correlated with the flow cytometric analysis where MHC-II expression is decreased by ∼50% in the CD11b^+^ LN cells from infected B6.WT mice compared to uninfected mice (Figure S3A). MHC-II downregulation was observed in both resistant and susceptible mice (data not shown). Figure S3.B illustrated representative serial sections where colocalization is seen for CD11b^+^, iNOS^+^, MHC-II^hi+^ and DsRed^+^ LN surfaces. Altogether, these results demonstrated that CD11b^+^ CD11c^+^ cells are by far the most abundant cells expressing iNOS, and are the most highly infected population of cells infected with *L. major*, within the LN at four weeks p.i. of B6.WT mice.

### *In situ* characterization of iNOS-producing cells in the cutaneous lesion

**Figure 4:**
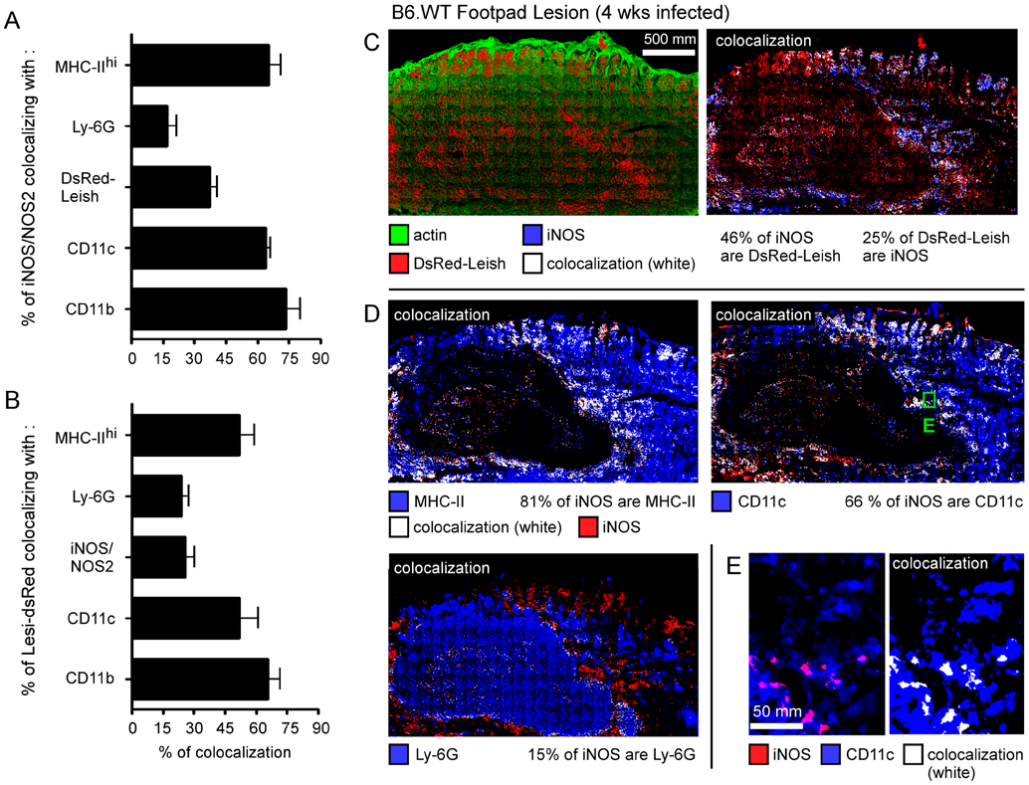
Characterization of iNOS-expressing cells in infected footpad. Wild type C57BL/6 mice were injected into the footpad with PBS or DsRed expressing *L. major* promastigotes (DsRed-Leish). Mice were sacrificed four weeks post-infection, footpad were collected and examined by immunofluorescence microscopy. *A*, The percentage of iNOS^+^ cells that colocalize with MHC-II^hi^-, Ly-6G-, CD11c-, CD11b-expressing cells and DsRed-Leish. *B*, The percentage of DsRed^+^ cells that colocalize with MHC-II^hi^-, Ly-6G-, iNOS-, CD11c- and CD11b-expressing cells. The bars are the mean±SD from at least 3 LN sections per LN from 8 mice. *C–E*, Serial footpad sections were analyzed for actin, DsRed, iNOS, MHC-II, CD11c and Ly-6G expression. Panels are color-coded with the text for the antigen or DsRed-Leish examined as well as the colocalization. Numbers in Figure 4D indicate the percentage of colocalizing cells in the upper panel. *E*, Higher magnification view of Figure 4D. Scale bar = 500 and 50 µm, as indicated. Data are representative of 3 independent experiments.

Few studies have investigated the phenotype of iNOS^+^ cells [7] or infected cells [28] in the *L. major*-induced cutaneous lesion. Using the same immunofluorescence microscopy technique developed for the analysis of the LN, we investigated which cells were infected in the cutaneous lesion of the B6.WT footpad four weeks p.i. As in the draining LN, the majority of the iNOS staining colocalized with CD11b (∼75%), CD11c (∼60%), MHC-II^hi^ (∼60%) and DsRed signal (∼40%) (Figure 4A). The surface occupied by CD11c^+^ iNOS^+^ staining corresponds to 3.55%+/−0.9% (mean of eight lesion sections) of the total section surface, which is determined by actin staining. We also observed that most of the DsRed-Leish^+^ surfaces colocalized with CD11b (∼65%), CD11c (∼55%) and MHC-II^hi^ (∼55%), whereas only 20–25% colocalized with Ly-6G and iNOS (Figure 4B). The Figures 4C–E showed representative examples of colocalization between CD11c^+^, iNOS^+^, MHC-II^+^ and DsRed^+^ signals in serial footpad sections. Figure S4A and S4B depicted colocalization between DsRed-Leish^+^ surface and CD11b^+^, CD11c^+^, MHC-II^+^ and Ly6G^+^, or MHC-II, CD11c and Ly-6G in the footpad, respectively. Together, these analyses revealed that (i) a very similar pattern of expression exists for CD11c and MHC-II (ii) the majority of the Ly-6G^+^ area does not colocalize with CD11c or MHC-II and (iii) CD11b^+^ is expressed by CD11c^+^/MHC-II^+^/Ly-6G^−^ (DC) and CD11c^−^/MHC-II^−^/Ly-6G^+^ (granulocytes) populations. In summary, these data revealed for the first time that inflammatory DC are the most abundant cell type expressing iNOS, whereas inflammatory DC and granulocytes are the most commonly infected cell type within the infected footpad of B6.WT mice 4 weeks p.i.

### Characterization of factors affecting the frequencies of iNOS-producing inflammatory DC

The lesion size during *L. major* infection was monitored in B6.WT, B6.TLR2/4, B6.TLR9, B6.MyD88, B6.TRIF mice and BC.WT mice. Our group and others have previously observed that MyD88 adaptor molecule [8], and to a lesser extent TLR9 [29],[30], are critical innate sensing molecules that promote resistance to *L. major* in B6.WT mice (Figure S5A and S5B). Immunofluorescent microscopy was utilized to examine the levels of DsRed expressing *L. major* and to determine whether the size of the lesion directly correlated with increased *L. major* replication within the footpad. Indeed, the DsRed-Leish^+^ surface per footpad sections in B6.TLR9 (19.52%) and B6.MyD88 (24.91%) mice was increased when compared to B6.WT (8.81%) mice (Figure S5C). Statistical analysis of these sections further confirmed this correlation (Figure S5C) and also suggested a negative role of TRIF in controlling *L. major* growth. In turn, the same analysis performed on the infected draining LN also highlighted a key role for MyD88 and TLR9 as well as a minor contribution of TLR2/4 in the control of *L. major* burden (Figure S5.E). Similar results were obtained when we determined the number of parasites per infected LN using a limit dilution assay (Figure S6). MyD88^−/−^ and to a lesser extent TLR9^−/−^ mice showed the higher level of living parasites. TLR2/4^−/−^ mice displayed a slightly higher, but significant, level of living parasite per LN when compared to WT mice. TLR2^−/−^ and TLR4^−/−^ mice did not present any enhanced parasite count (data not shown). We also excluded a possible contribution of the MyD88-dependent inflammatory pathway as infected IL-18, IL-1beta-converting enzyme (ICE)-deficient mice did not exhibit increased parasite count compared to infected B6.WT.

**Figure 5:**
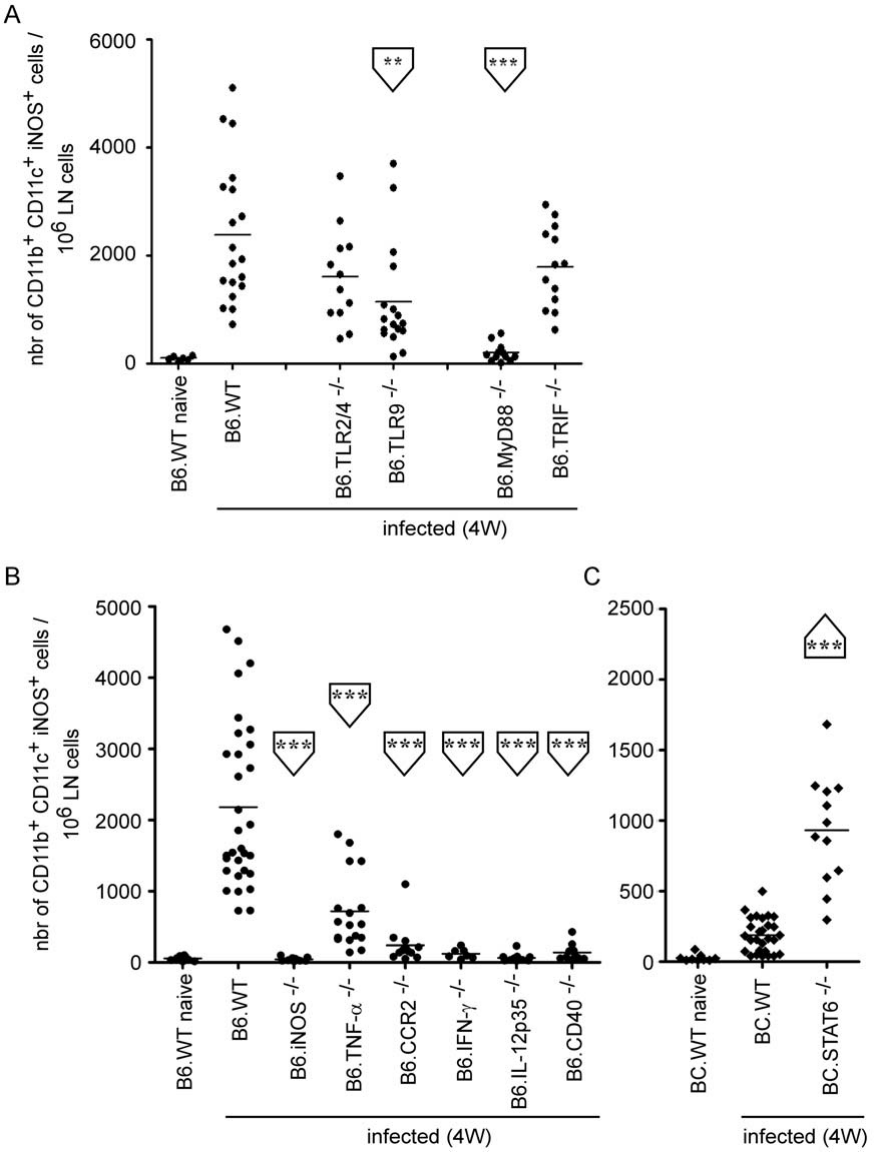
Regulation of iNOS-producing DC activation following *Leishmania* infection. Wild-type, TLR2/4-, TLR9-, MyD88-, TRIF-, iNOS-, TNF-α-, CCR2-, IFN-γ-, IL-12p35- and CD40-deficient C57BL/6 as well as wild type and STAT6-deficient BALB/c mice (at least 4 mice per group) were injected into the footpad with PBS or *L. major* promastigotes. *A–C*, The number of CD11b^+^ CD11c^+^ iNOS^+^ cells per 10^6^ LN cells acquired. Each data point represents the value obtained from an individual LN and the data are pooled from 2 independent experiments. Student's *t* test was performed where *, **, and *** denote significance of *p*<0.05, *p*<0.01, and *p*<0.001, respectively, compared to infected B6.WT (A–B) and BC.WT (C).

Next, we investigated whether TLR9-MyD88 signaling was linked to iNOS production by inflammatory DCs. B6.TLR9 and B6.MyD88 mice, but not B6.TLR2/4 and B6.TRIF mice, showed statistically significant reductions in the number of iNOS^+^ CD11b^+^ CD11c^+^ cells when compared to B6.WT mice, suggesting that *L. major*-derived PAMPs detected by this innate-sensing pathway lead directly to iNOS production by inflammatory DC (Figure 5A).

For decades, immune control of *L. major* infection has been associated with the development of a Th1-mediated response in B6.WT mice, and the production of IFN-γ by CD4^+^ T cells [1],[2]. In contrast, the Th2 cytokine profile (i.e. IL-4 and -13) observed in *L major* infected, susceptible BC.WT is promoted through a STAT6-dependent signaling pathway [15]. Therefore, we examined the importance of various factors, i.e. cytokines and chemokines, implicated in the establishment of protective Th1 responses and playing a role in cellular recruitment, to determine which pathways might regulate the recruitment of iNOS^+^ CD11b^+^ CD11c^+^ cells to the infected draining LN. We found that IL-12p35, CD40, IFN-γ and CCR2 expression were absolutely required for recruitment/development of iNOS-expressing inflammatory DC in the infected LN, while TNF-α played a less important role in this process (Figure 5B). Consistent with these results, STAT6-deficient BALB/C (BC.STAT6) mice, which are defective in IL-4 and IL-13 signaling, showed higher levels of iNOS^+^ CD11b^+^ CD11c^+^ cells, when compared to infected BC.WT mice (Figure 5C).

These observations suggest that the presence of iNOS^+^ inflammatory DC in the infected LN is largely dependent upon the development of an IFN-γ-mediated Th1 protective response against *L. major* infection. In agreement with this hypothesis, we observed a statistical reduction in the frequency of IFN-γ^+^ TCRβ^+^ CD4^+^ LN T cells in B6.TLR2/4, B6.TLR9, B6.MyD88, B6.CCR2, B6.IL-12p35 and B6.CD40 mice when compared to B6.WT mice (Figure S7A–C). Moreover, BC.STAT6 mice displayed an increased number of IFN-γ-producing CD4^+^ T cells compared to BC.WT mice (Figure S7.D).

In summary, these observations strongly suggest that the resistance to *L. major* infection is closely associated with the presence of iNOS-producing inflammatory DC, which seems dependent on the development of a Th1 microenvironment by IFN-γ-producing CD4^+^ T cells.

### Identification of factors regulating the recruitment of inflammatory DC to infected draining lymph node

**Figure 6:**
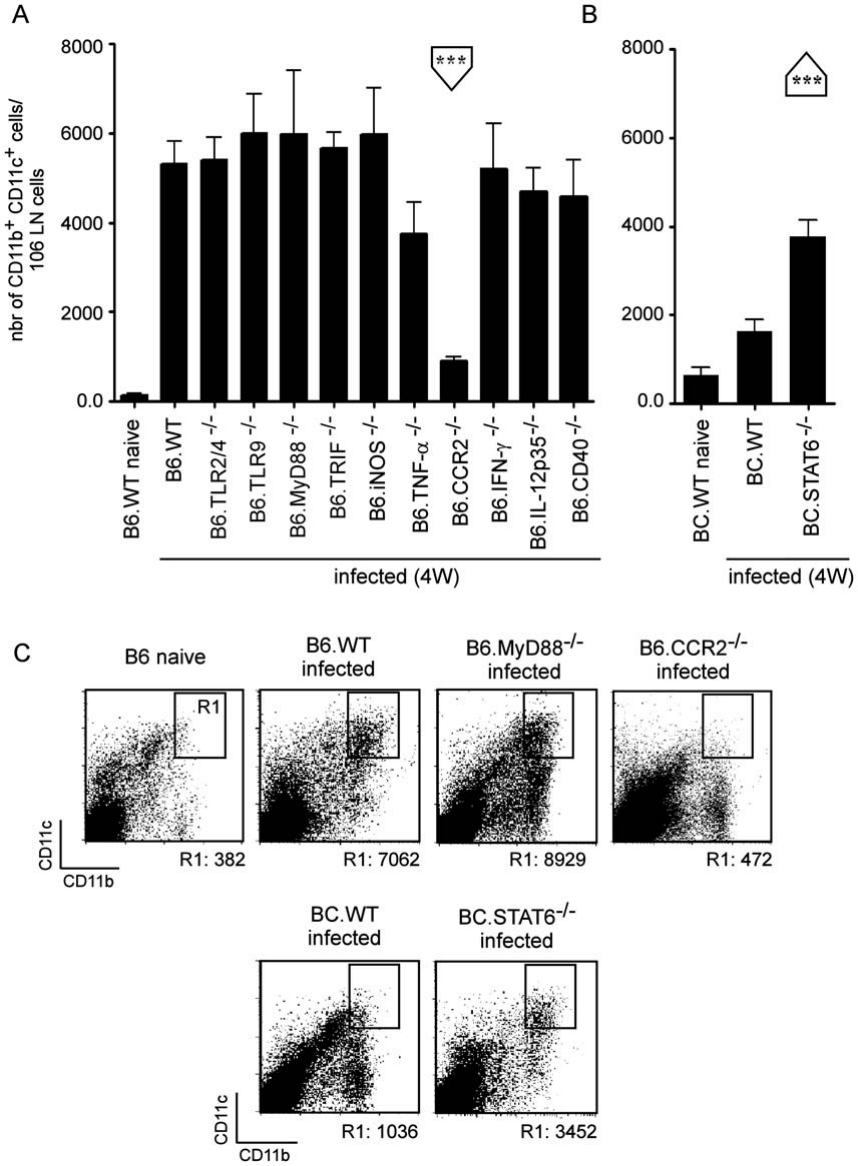
CCR2-dependent recruitment of iNOS-producing DC recruitment following *Leishmania* infection. Wild-type, TLR2/4-, TLR9-, MyD88-, TRIF-, iNOS-, TNF-α-, CCR2-, IFN-γ-, IL-12p35- and CD40-deficient C57BL/6 as well as wild type and STAT6-deficient BALB/c mice (4 mice per group) were injected into the footpad with PBS or *L. major* promastigotes. Mice were sacrificed four weeks post-infection and the draining popliteal lymph nodes were collected and analyzed by flow cytometry. *A–B*, Bars show the mean±SD of the number of CD11b^+^ CD11c^+^ iNOS^+^ cells per 10^6^ LN cells acquired from at least 4 mice per group, and the data are pooled from 2 independent experiments. Student's *t* test was performed where * and *** denote significance of *p*<0.05 and *p*<0.001, respectively, between the indicated groups. *C*, Total pooled lymph node cells iNOS^+^-gated cells from uninfected and infected mice were analyzed for CD11b and CD11c expression. Results are derived from at least *n* = 4 mice and the data are representative of 3 independent experiments. R1 and R2 associated values correspond to the numbers of CD11b^hi^ CD11c^hi^ cells per 10^6^ cells among total LN and iNOS^+^ cells in the upper panels, respectively.

To further dissect the mechanism for iNOS-mediated control of *L. major* infection, we examined whether the factors that were required to promote increased frequencies of iNOS-producing inflammatory DC in the draining LN functioned at the level of recruitment only, or whether they might directly induce iNOS production once the cells were present in the infected LN. We found that only CCR2 deficiency decreased the frequency of the CD11b^+^ CD11c^+^ cells in infected LN (Figure 6A), while in turn STAT6 deficiency in susceptible BC.WT favored their recruitment (Figure 6B). Figure 6C depicts representative flow cytometric analyses, summarizing the role of specific factors implicated in the recruitment of CD11c^+^ CD11b^+^ DC (R1 gate).

## Discussion

Reactive oxygen intermediates (ROI) and reactive nitrogen intermediates (RNI) can damage DNA and several chemical moieties necessary for the replication/division of both host cells and pathogens, and their production constitutes an essential arm of the immune response to microbial infections. High level production of ROI and RNI is typical of infected phagocyte cells, including granulocyte and monocyte-derived cells [4]. ROI and RNI production seems to be largely redundant, as illustrated by the fact that gp91^phox−/−^ (ROI-deficient) and iNOS/NOS2^−/−^ (RNI-deficient) mice are viable in normal housing conditions [4]. However, in the case of *Leishmania* infection, gp91^phox−/−^ C57BL/6 mice largely control *Leishmania* growth, whereas iNOS^−/−^ C57BL/6 mice display a quite dramatic phenotype during the first weeks of infection [5],[6],[7]. Lesions in iNOS^−/−^ mice appear highly necrotic 3–4 weeks after *L. major* infection ([5],[6],[7] and Figure S1) and mutilation is observed before 5 weeks. In comparison, TNF-α^−/−^ [25],[28], MyD88^−/−^ [8], IL-12^−/−^ [10] or IFN-γ^−/−^ [11] C57BL/6 mice, normally thought of as highly susceptible mice, display necrotic lesions only after 4 or 6 weeks of infection and mutilation is observed only after 8–10 weeks. These observations demonstrate that among Th1 effectors, RNI constitutes a non redundant and crucial immune mechanism for control of *L. major* growth. This is also substantiated by the fact that *L. major* infection can be reactivated in chronically infected healthy C57BL/6 mice following iNOS inhibitor treatment [7]. To this point, cells expressing iNOS *in vivo* during *L. major* infection have been only generally characterized [7] as macrophages and dendritic cells (DCs) based on their expression of F4/80 and NLDC-145 markers, respectively.

DCs were originally described as the population of splenocytes which were responsible for promoting the mixed lymphocyte reaction. Such splenic DC, known as “conventional” DC, cDC, are present in all lymphoid organs and are essential for the induction of immunity [19],[20]. However, the term “DC” now refers to a group of several cell populations in addition to cDCs that differ in their cellular origin, their localization and their role in immune response [23]. The antigen-presenting cell (APC) function of nearly all DC populations seems to remain their main general characteristic. Among DC subsets, inflammatory DCs (also termed TNF-α iNOS-producing DC, TipDC) produce TNF-α, nitric oxide (NO), IL-12 and can stimulate T cells [18],[22]. They are mainly defined by the expression of CD11b, CD11c, CD115, MHC-II and Ly-6C markers and most likely derived from CD11b^+^ CD11c^−^ CD115^+^ Ly-6C^+^ “inflammatory” monocytes that are recruited to inflamed tissues, spleen and lymph nodes [23]. They represent the major source of iNOS in the spleen from *Listeria monocytogenes* [22] and *Brucella melitensis* [24] infected mice. A recent work by the group of C. Ardavin [18] has reported that cells expressing the cell surface phenotype of inflammatory DC were recruited in skin lesions and draining lymph nodes of *L. major* infected mice. In our study, we formally demonstrated that these inflammatory DC are the main source of iNOS protein (70–75% of total iNOS-producing cells on tissue section and more than 90% by flow cytometry analyses) and, therefore, potentially represent a key effector cells for the defence to *L. major* infection. Flow cytometry analyses of draining lymph node cells from C57BL/6 infected mice showed that 80–90% of iNOS^+^ cells express the typical phenotype of inflammatory DC: CD4^−^ CD8α^−^ CD11b^+^ CD11c^+^ CD40^+^ CD62L^−^ CD115^+^ F4/80^+^ 7/4^+^ Ly-6C^+^ Ly-6G^−^ Mac3^+^ and MHC-II^+^. In agreement with this previous result, we confirmed that 60 to 70% of iNOS^+^ cells detected in tissue sections express CD11c^+^ and CD11b^+^ using immunohistofluorescence techniques and colocalization analyses. In comparison, granulocytes identified by Ly-6G expression represent only 10–15% of iNOS^+^ cells. Interestingly, the frequency of inflammatory DC expressing detectable level of TNF-α protein appears very small when compared to the frequency of iNOS^+^ cells, 2–6% and 30–40%, respectively (Figure 1.C). Among iNOS-producing inflammatory DC, the frequency of TNF-α^+^ cells is 0.5–2%, suggesting that in *L. major* model, like in *B. melitensis* model [24], inflammatory DC can be mainly characterized by their iNOS production.

Despite advances made in mouse models of *L. major* infection, many parameters regarding the nature and cell surface phenotype of infected cells remain poorly characterized. Initially, *L. major*-infected cells were largely thought to be macrophages. However, we reported that cells expressing high level of CD11c, a DC specific characteristic, are the most frequently infected cells in the draining lymph nodes of infected mice [28]. In the work from the group of C. Ardavin [18], DsRed-expressing *L. major* has been used for flow cytometric characterization of infected cells in the lesions and draining lymph nodes. Monocytes (CD11b^+^ CD11c^−^ F4/80^int^ Ly-6C^high^), macrophages (CD11b^+^ CD11c^−^ F4/80^high^ Ly-6C^int^) and inflammatory DC (CD11b^+^ CD11c^+^ F4/80^int^ Ly-6C^high^) were found infected. However, this approach did not exclude the possibility that during purification (i) non infected phagocytic cells might become infected *in vitro*, (ii) extracellular parasite adheres to cells or (iii) that cells infected *in vivo* might be lost *ex vivo*. Using flow cytometric analyses, we have detected the presence of “false-positive” DsRed-expressing cells in LN cells isolated from infected mice. This demonstrated that the manipulation of infected cells/tissues *ex vivo* can result in the infection of cells or adherence of parasite to cells that are normally not infected *in situ*. In agreement, when cytospins were performed on sorted DsRed-Leish^+^ cells from infected mice, we observed that a fraction of these cells do not show intracellular presence of parasite, but just display the parasite bound to their cell surface. Consequently, these very important technical considerations led us to restrict our analysis about the cellular tropism of *L. major* infection in tissues and draining LN to *in situ* analysis. We observed that 70–80% of DsRed signal colocalized with CD11b and CD11c inflammatory DC markers in tissue section from draining lymph node of C57BL/6 mice while only 20–30% costained with Ly-6G granulocyte marker and less than 5% with B220 marker (used as negative control). More interestingly, 30–40% of iNOS^+^ signal overlap with DsRed and 70–80% of DsRed signal with iNOS. In lesion tissue sections, the phenotype of DsRed cells appeared similar to that observed in the draining lymph node with the exception of the iNOS marker. Only 20–30% of DsRed signal colocalized with iNOS. This difference could be explained by the very high level of DsRed signal found in lesion (5–10% of DsRed^+^ surface among lesion surface) when compared to draining lymph node (0.01–0.1% of DsRed^+^ surface among lymph node surface). In total, these data demonstrate that inflammatory DCs are oftentimes infected *in vivo* by *L. major*, the iNOS^+^ subset being the most frequent of these and constituting the major infected cell population in draining lymph node.

Recent studies in *L. monocytogenes* [31] and *T. gondii* [32] model have shown that Ly-6C^high^ inflammatory monocyte recruitment to sites of infection involved CCR2-mediated emigration of monocytes from the bone marrow into the bloodstream. In agreement, we also observed a drastic inhibition of inflammatory DC recruitment into the draining lymph node of CCR2^−/−^ C57BL/6 mice in the *L. major* model. Factors regulating the activation of effector functions of inflammatory DCs *in vivo* remain largely undetermined. *In vitro* studies have shown that regulation of iNOS gene expression is very complex. The murine iNOS gene promoter contains nearly 30 consensus binding sites for known transcriptional factors [33],[34]. In the *L. monocytogenes* model, iNOS production by inflammatory DCs appeared MyD88 dependent [35]. In our *L. major* model, we observed a close association between susceptibility to infection and reduced iNOS production by inflammatory DCs. BALB/c susceptible mice displayed decreased recruitment and activation of inflammatory DCs when compared to resistant C57BL/6 mice. We took advantage of this model to try to identify important factors for regulating iNOS expression by inflammatory DCs. A defect in iNOS production, but not in recruitment, for inflammatory DC was observed in C57BL/6 mice deficient for MyD88, TLR9, CD40, IL-12, IFN-γ and TNF-α. In contrast, in STAT-6^−/−^ BALB/c mice, that are defective for IL-4 and IL-13 signal transduction, the frequency of iNOS-producing inflammatory DCs is clearly enhanced when compared to wild-type BALB/c mice. In summary, these results demonstrated that Th1 and Th2 responses have opposite effect on effector function of inflammatory DC. Deficiencies in IFN-γ or factors affecting its production (e.g. CD40, IL-12, and MyD88) in C57BL/6 mice negatively affect the frequency of iNOS-producing DC. As IFN-γ is mainly produced in our model by CD4^+^ T cells, this suggests that these cells have an important role in the regulation of inflammatory DC. Interestingly, the study from C. Ardavin group suggests that inflammatory DC could be responsible to the Th1 differentiation of CD4^+^ T cells during *L. major* infection because they produce IL-12 and display *L. major*-derived antigens associated to MHC-II molecules [18]. Thus, their data as well as ours suggest a positive cross-regulation between inflammatory DCs and CD4^+^ T cells during *L. major* infection. iNOS production by inflammatory DCs also required TNF-α as demonstrated by the fact that TNF-α^−/−^ C57BL/6 mice display reduced frequency of iNOS-producing inflammatory DCs, despite of an extremely high frequency of IFN-γ-producing CD4^+^ T cells. On the contrary, neutralisation of Th2 responses enhances iNOS-expressing inflammatory DC frequency in BALB/c mice. These observations are supported by several *in vitro* studies on established cell lines showing that iNOS gene expression is positively regulated by IFN-γ [36] and TNF-α [37] and negatively regulated by IL-4 [36] and IL-13 [38].

In summary, our study showed a strong association between the recruitment and activation of inflammatory DC and the resistance to *L. major*. In addition, we showed that iNOS production by inflammatory DCs is positively regulated by Th1 response and negatively by Th2 response. Taken together, our results provide new insight into how innate and adaptive immune responses fight *L. major* infection. A better understanding of the mechanisms regulating inflammatory DC recruitment and activation could lead to new therapeutic strategies against *Leishmania* infection.

## Materials and Methods

### Mice and parasites

Genetically deficient mice in C57BL/6 background: TLR2/4^−/−^ mice from Dr. T. van der Poll (Academic Medical Center, The Netherlands), TLR9^−/−^ [39] and MyD88^−/−^ [40] were obtained from Dr. S. Akira (Osaka University, Japan). TRIF^−/−^ mice [41] were a kind gift from Dr. B. Beutler (The Scripps Research Institute, CA), TNF-α^−/−^ mice [42] from Dr. S. Magez (Vrije Universiteit Brussel, Belgium), IL-12p35^−/−^ mice [10] from Dr. B. Ryffel (University of Orleans, France), iNOS mice [6] from Dr. G. Lauvau (Université de Nice-Sophia Antipolis, France), IFN-γ^−/−^ mice [43] from Dr. M. Moser (Université Libre de Bruxelles, Belgium), CCR2^−/−^ mice [44] from Dr. G. Brusselle (Universitair Ziekenhuis Gent, Belgium). STAT-6^−/−^ BALB/c mice [45] were obtained from The Jackson Laboratory (Bar Harbor, ME). Wild type C57BL/6 mice and BALB/c mice, purchased from Harlan (Bicester, UK), were used as control. All mice used in this study were bred in the animal facility of the Free University of Brussels (ULB, Belgium). The maintenance and care of mice complied with the guidelines of the ULB Ethic Committee for the use of laboratory animals.

*Leishmania major* promastigotes (World Health Organization strain WHOM/IR/-/173) were grown in M199 medium containing 20% FCS. Discosoma Red (DsRed) Protein expressing promastigotes [27] were selected as previously described [46].

### Mice infection

*Leishmania major* parasites were harvested in stationary phase after 6 to 8 days of culture growth, centrifuged (2,500 rpm, 10 min, 20°C) and washed in PBS (buffer). Promastigotes were purified by 10% Polysucrose (Sigma) gradient and washed three times in PBS before being used for infection. Mice were infected s.c. in the hind footpad with 10^6^ promastigotes in a final volume of 25 µl. The thickness of infected footpads was weekly monitored with a metric caliper (in mm; Kroeplin, Schlüchtern, Germany). Mice were killed at indicated times by cervical dislocation. Footpad lesions (cut tangentially to the bone ground) and popliteal draining lymph nodes were collected for cytofluorometric and microscopic analyses. Tissue parasite burden was determined by limiting dilution analysis

### Cytofluorometric analysis

Popliteal draining lymph nodes were harvested and digested with a cocktail of DNAse I fraction IX (Sigma-Aldrich Chimie SARL, Lyon, France) (100 µg/ml) and 1.6 mg/ml of collagenase (400 Mandl U/ml) at 37°C for 30 min. After washing, lymph node cells were filtered and first incubated in saturating doses of purified 2.4G2 (anti-mouse Fc receptor, ATCC) in 200 µl PBS 0.5% BSA 0.02% NaN3 (FACS buffer) for 10 minutes on ice to prevent antibody binding to Fc receptor. 3–5×10^6^ cells were stained on ice with various fluorescent mAbs combinations in FACS buffer and further collected on a FACScalibur cytofluorometer (Becton Dickinson, BD). We purchased the following mAbs from BD Biosciences: Biotin-coupled 53-2.1 (anti-CD90.2), AFS98 (anti-CD115), AL-21 (anti-Ly-6C), M5/114.15.2 (anti- IA/IE), 3/23 (anti-CD40), Fluorescein (FITC)-coupled OX-7 (anti-CD90.1), 1A8 (anti-Ly-6G), RM4-5 (anti-CD4), 53-6.7 (anti-CD8α), M1/70 (anti-CD11b), Phycoerythrin (PE)-coupled HL3 (anti-CD11c). Allophycocyanin (APC)-coupled BM8 (anti F4/80). Biotin-coupled 7/4 (anti-neutrophil) was obtained from Caltag Laboratories. Biotin-coupled mAbs were stained with FITC or PE-coupled streptavidin from BD Biosciences. The cells were analyzed on a FACScalibur cytofluorometer. Cells were gated according to size and scatter to eliminate dead cells and debris from analysis.

### Intracellular cytokine staining

Lymph node cells were treated as previously described [24]. Lymph node cells were incubated for 4 h in RPMI 1640 5%FCS with 1 µl/ml Golgi Plug (BD Pharmingen) at 37°C, 5%CO2. The cells were washed with FACS buffer and stained for cell surface markers before fixation in PBS/1% PFA for 15–20 min on ice. These cells were then permeabilized for 30 min using a saponin-based buffer (1× Perm/Wash, BD Pharmingen in FACS buffer) and stained with one or a combination of the following intracellular mAbs: Phycoerythrin-coupled M3/84 (anti-Mac3; BD Biosciences), Phycoerythrin-coupled MP6-XT22 (anti-TNF-α; eBioscience), allophycocyanin-coupled MP6-XT22 (anti-TNF-α; BD Biosciences), allophycocyanin-coupled XMG1.2 (anti-IFN-γ; BD Biosciences), purified M-19 (rabbit polyclonal IgG anti-NOS2; Santa Cruz Biotechnology) stained with Alexa Fluor 647 goat anti-rabbit (Molecular Probes). After final fixation in PBS/1% PFA, cells were analyzed on a FACScalibur cytofluorometer. No signal was detectable with control isotypes.

### Histochemical staining on cytospin

Draining lymph node cells from four weeks infected mice were washed 3 times in PBS, and spun down onto glass slides. Slides were air-dried overnight, fixed in acetone, stained with hematoxylin/eosin (Vector Laboratories Inc., Burlingame, CA) and dehydrated in ethanol series. Slides were mounted and digitized image were captured using Zeiss inverted microscope (Axiovert 200) equipped with high resolution monochrome camera (AxioCam HR, Zeiss).

### Immunofluorescence microscopy

Footpad lesions and lymph nodes were fixed for 3 h at 4°C in 1% paraformaldehyde (pH 7.4), washed in PBS, incubated overnight at 4°C in a 20% PBS-sucrose solution under agitation, and washed again in PBS. Tissues were embedded in the Tissue-Tek OCT compound (Sakura), frozen at −80°C, and cryostat sections (10 µm) were prepared. Tissues sections were rehydrated in PBS, then incubated successively in a PBS solution containing 1% blocking reagent (Boeringer) (PBS-BR 1%) and in PBS-BR 1% containing Alexa Fluor 488 phalloidin (Molecular Probes) and any of the following mAbs: purified 1A8 (anti-Ly-6G), or rabbit polyclonal antibodies anti-NOS2 (Calbiochem) (note that M-19 anti-NOS2; used for cytofluorometric analysis is not use for immunofluorescence microscopy), biotin-coupled M1/70, HL3 and RA3-6B2 (anti-CD45R/B220, BD Biosciences) as well as APC-coupled BM8 and M5/114.15.2 Uncoupled 1A8 mAb and anti-NOS2 polyclonal antibodies were detected using biotin-coupled R67/1.30 (mouse anti-rat IgG2a, BD Biosciences) and Alexa Fluor 647-coupled goat anti-rabbit IgG (Molecular Probes) in PBS-BR 1%, respectively. Biotin-coupled mAbs were amplified using Alexa Fluor 350 or Alexa Fluor 647 Streptavidin (Molecular Probes) in PBS-BR 1%. When two biotin-coupled mAbs were used, free biotin sites were saturated with an avidin-biotin blocking kit (Vector). Slides were mounted in Fluoro-Gel medium (Electron Microscopy Sciences, Hatfield, PA). Labeled tissues sections were visualized under a Zeiss fluorescent inverted microscope (Axiovert 200) equipped with high resolution monochrome camera (AxioCam HR, Zeiss). All images were acquired with 63× objective at maximal camera resolution. Acquisition of entire tissue section surface by automatic scanning and measurement of colocalization between two staining was realized using MosaiX module and Colocalization module, respectively, from AxioVision program (Zeiss). When images were treated with The Colocalization module, double positive surface was stained in white (as indicated in Figures).

### Statistical analysis

We have used a (Wilcoxon-) Mann-Whitney test provided by GraphPad Prism program to statistically analyze our results. Each group of deficient mice was compared to wild type mice. We also compared each group to each other and displayed the result when it is required. Values of p<0.05 were considered to represent a significant difference. *, **, *** denote p<0.05, p<0.01, p<0.001, respectively.

## Supporting Information

Figure S1 iNOS-deficient mice are highly susceptible to *L. major* infection. *A*, Visualisation of the footpad from wild type, iNOS-, TNF-α-deficient C57BL/6 and wild type BALB/c mice injected with PBS or *L. major* promastigotes. *B*, Size of footpad during the course of *L. major* infection in same groups of mice. Results are expressed as means±SD from at least *n* = 6 mice per group and the data are representative of 3 independent experiments.
(8.37 MB TIF)

Figure S2 False positive signals generated after DsRed *Leishmania* infection using cytofluorometric analyses. CD90.1 and CD90.2 congenic wild-type BALB/c mice were injected into the footpad with PBS or DsRed-expressing *L. major* promastigotes, respectively. Mice were sacrificed four weeks post-infection and the draining popliteal lymph nodes were collected. *A*, Total pooled lymph node cells from uninfected and infected CD90.2 BALB/c mice as well as DsRed-Leish^+^ cells (R1 gate) were analyzed by flow cytometry for FSC, CD3ε, IgD, CD11b and CD11c expression. *B*, Uninfected CD90.1, infected CD90.2 and a mix of uninfected CD90.1 and infected CD90.2 LN cells were analyzed for DsRed signal by flow cytometry. Total lymph node cells as well as DsRed-Leish^+^-gated cells (R1) were analyzed for CD90.1 and CD90.2 expression. *C*, Draining lymph node cells from four weeks infected mice were washed 3 times in PBS, spun down onto glass slides and stained with hematoxylin/eosin. Pictures represent the resulting cytospins of infected LN cells showing various forms and degree of infection. Red arrow represented a parasite bound to the extracellular membrane of a non infected cells purified from draining LN of infected mice.
(0.80 MB TIF)

Figure S3 Downregulation of MHC-II expression following *L. major* infection. Wild-type C57BL/6 mice were injected into the footpad with PBS or DsRed-expressing *L. major* promastigotes. Mice were sacrificed four weeks post-infection and the draining popliteal lymph nodes were collected. *A*, Total lymph node cells were analyzed for MHC-II, iNOS and CD11b expression by flow cytometry. *B*, Serial LN sections were analyzed for CD11b, actin, DsRed and iNOS expression by immunofluorescence. Panels are color-coded with the text for the antigen or fluorescent *L. major* parasite examined as well as the colocalization. Numbers indicate the percentage of colocalizing cells in the upper panel. Scale bar = 400 µm. Data are representative of 3 independent experiments.
(2.66 MB TIF)

Figure S4 Characterization of infected cells in footpad lesion. C57BL/6 mice were injected into the footpad with PBS or DsRed-expressing *L. major* amastigotes. Mice were sacrificed four weeks post-infection, footpad were collected and examined by immunohistochemistry. *A–B*, Serial footpad sections were analyzed for actin, DsRed, CD11b, CD11c, MHC-II and Ly-6G expression. Panels are color-coded within the text for the antigen or fluorescent *L. major* parasite examined as well as the colocalization. Numbers indicate the percentage of colocalizing cells in the upper panel. Scale bar = 500 µm. Data are representative of 3 independent experiments.
(2.24 MB TIF)

Figure S5 TLR-associated signalling pathways and susceptibility to *L. major* infection. Wild-type, TLR2/4-, TLR9-, MyD88- and TRIF-deficient C57BL/6 mice as well as wild type BALB/c mice (at least 8 per group) were injected into the footpad with PBS or DsRed-expressing *L. major* promastigotes (DsRed-Leish). *A–B*, Size of primary footpad lesions was analyzed during the course of *L. major* infection for each group of mice. Results illustrate one representative experiment performed with 8 animals of each strain and expressed as means±SD. 3 independent experiments have been performed. *C*, Naïve and infected wild type C57BL/6 mice as well as infected TLR9 and MyD88-deficient C57BL/6 mice were sacrificed four weeks post-infection, footpad (C–D) and LN (E) were collected and examined by immunofluorescence. Footpad (C–D) and LN (E) sections were analyzed for actin and DsRed expression. *C*, Panels are color-coded within the text for actin or DsRed-Leish. Numbers indicate the percentage of DsRed-Leish positive surface per footpad surface in the upper panel. *D–E*, Each data point represents the percentage of DsRed-Leish positive surface among footpad surface obtained from an individual footpad (D) or LN (E) and the data are pooled from two analyses. Student's *t* test was performed where *, **, and *** denote significance of *p*<0.05, *p*<0.01, and *p*<0.001, respectively, compared to infected wild type C57BL/6 mice.
(5.16 MB TIF)

Figure S6 TLR-associated signalling pathways and susceptibility to *L. major* infection. Wild-type, TLR2/4-, TLR9-, MyD88- and TRIF-deficient C57BL/6 as well as wild type BALB/c mice (at least 4 per group) were injected into the footpad *L. major* parasites. Each data point represents the number of parasites obtained from an individual LN and the data are pooled from two independent experiments. Student's *t* test was performed where ** and *** denote significance of *p*<0.01 and *p*<0.001, respectively, compared to infected wild type C57BL/6 mice.
(0.13 MB TIF)

Figure S7 Characterization of IFN-γ-producing cells following *Leishmania* infection. Wild-type, TLR2/4-, TLR9-, MyD88-, TRIF-, iNOS-, TNF-α-, CCR2-, IFN-γ-, IL-12p35- and CD40-deficient C57BL/6 as well as wild-type and STAT6-deficient BALB/c mice (at least 4 mice per group) were injected into the footpad with PBS or *L. major* promastigotes parasites. Mice were sacrificed four weeks post-infection and the draining popliteal lymph nodes were collected and analyzed by flow cytometry. *A*, Total lymph node cells were analyzed for FSC and IFN-γ expression. Total lymph node cells as well as IFN-γ-gated cells (R1) were analyzed for TCR-β and CD4 expression. Numbers in box indicate the number of positive cells per 10^6^ cells acquired total cells. *B–D*, Each data point represents the number of TCRβ^+^ CD4^+^ IFN-γ^+^ cells per 10^6^ LN cells acquired obtained from an individual LN and the data are pooled from two (B) or three (C–D) independent experiments. Student's *t* test was performed where *, **, and *** denote significance of *p*<0.05, *p*<0.01, and *p*<0.001, respectively, compared to infected B6.WT (B–C) or BC.WT (D).
(0.38 MB TIF)
