# Supplementary figures and images for: iNOS-Producing Inflammatory Dendritic Cells Constitute the Major Infected Cell Type during the Chronic Leishmania major Infection Phase of C57BL/6 Resistant Mice
Source: PLoS Pathog. 2009 Jun 26;5(6):e1000494. doi: 10.1371/journal.ppat.1000494 (PMC2695779; doi:10.1371/journal.ppat.1000494)

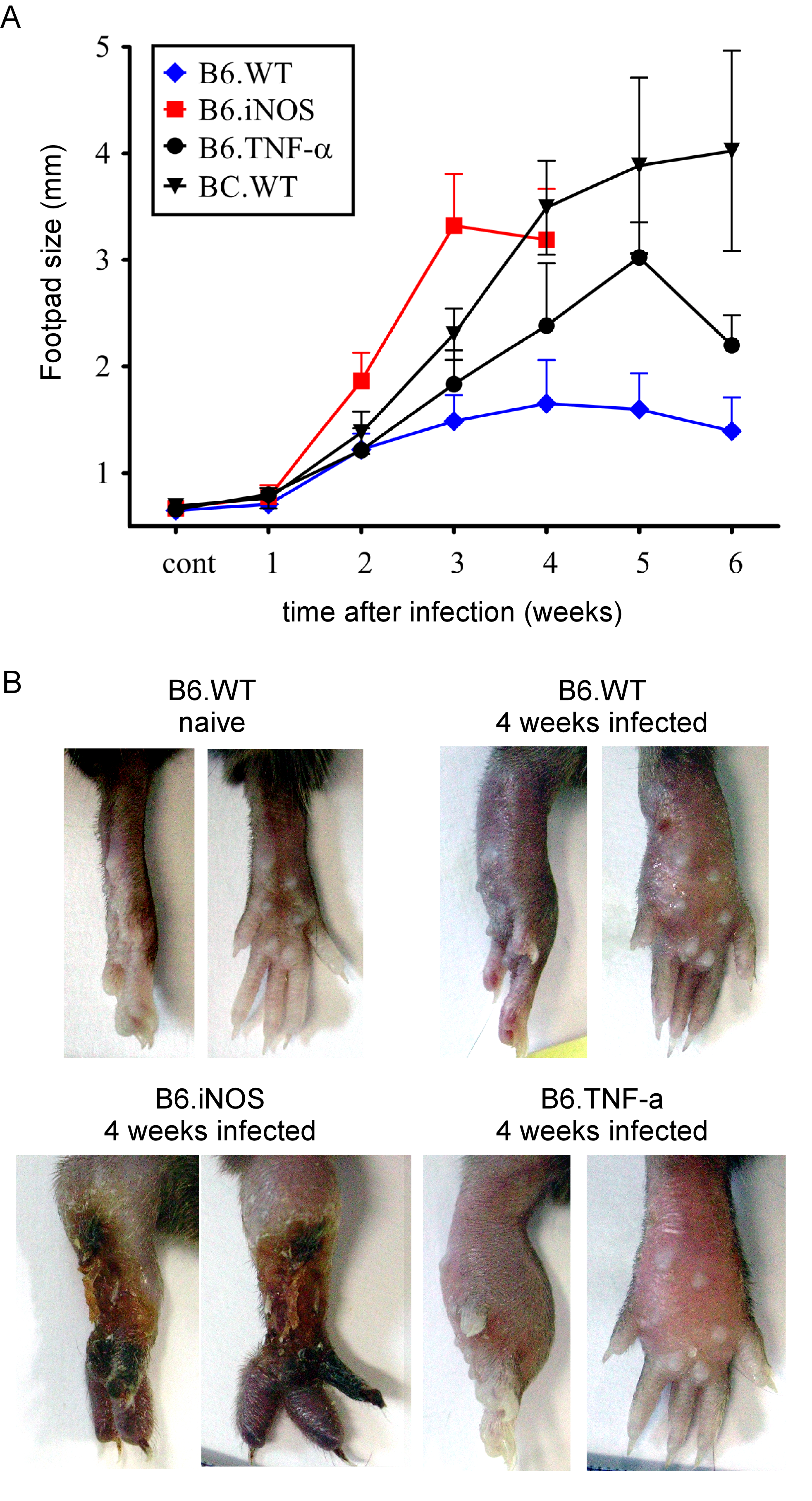

Supplement: Figure S1 — iNOS-deficient mice are highly susceptible to L. major infection. A, Visualisation of the footpad from wild type, iNOS-, TNF-α-deficient C57BL/6 and wild type BALB/c mice injected with PBS or L. major promastigotes. B, Size of footpad during the course of L. major infection in same groups of mice. Results are expressed as means±SD from at least n = 6 mice per group and the data are representative of 3 independent experiments. (8.37 MB TIF) [file ppat.1000494.s001.tif]

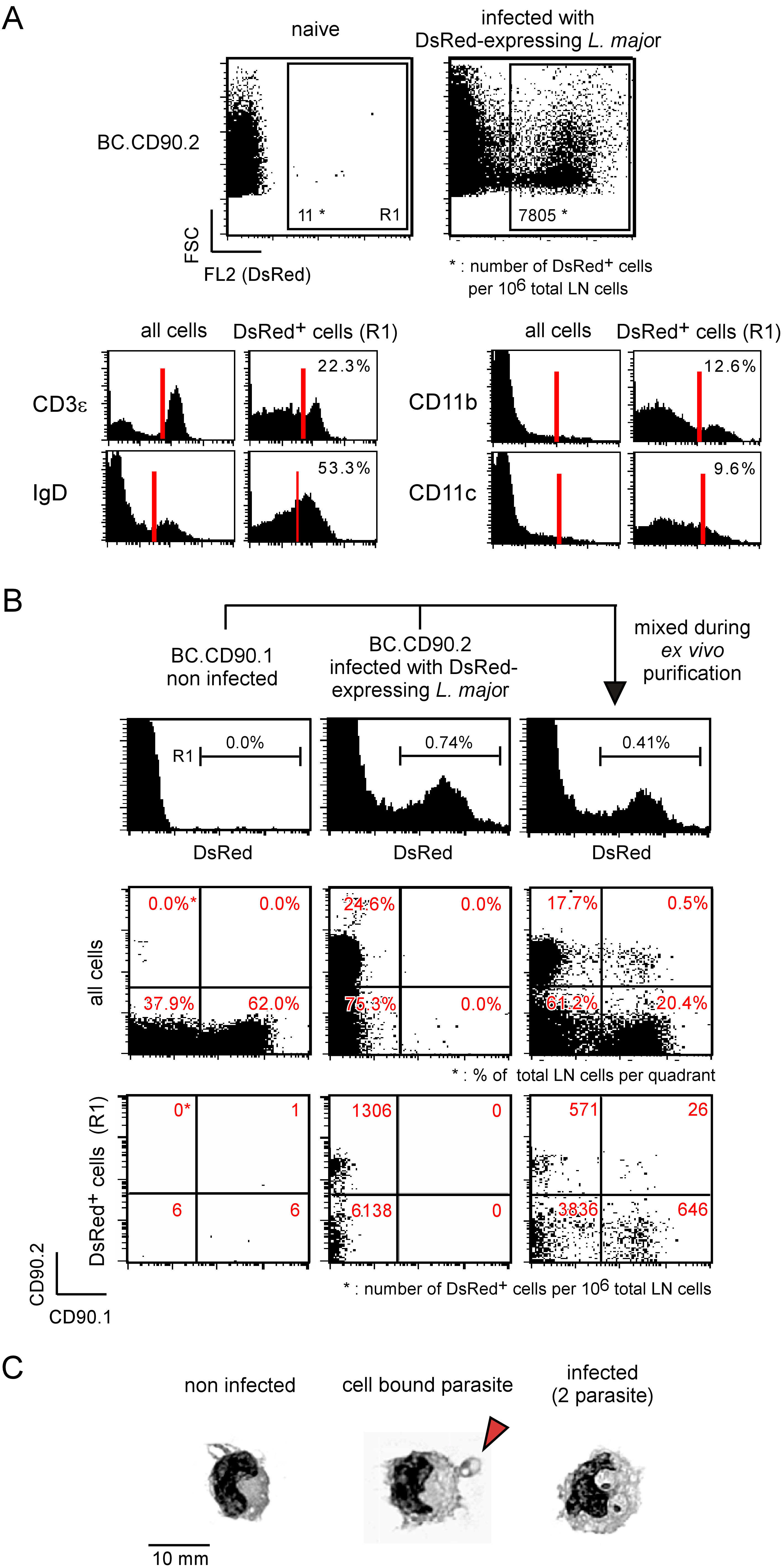

Supplement: Figure S2 — False positive signals generated after DsRed Leishmania infection using cytofluorometric analyses. CD90.1 and CD90.2 congenic wild-type BALB/c mice were injected into the footpad with PBS or DsRed-expressing L. major promastigotes, respectively. Mice were sacrificed four weeks post-infection and the draining popliteal lymph nodes were collected. A, Total pooled lymph node cells from uninfected and infected CD90.2 BALB/c mice as well as DsRed-Leish+ cells (R1 gate) were analyzed by flow cytometry for FSC, CD3ε, IgD, CD11b and CD11c expression. B, Uninfected CD90.1, infected CD90.2 and a mix of uninfected CD90.1 and infected CD90.2 LN cells were analyzed for DsRed signal by flow cytometry. Total lymph node cells as well as DsRed-Leish+-gated cells (R1) were analyzed for CD90.1 and CD90.2 expression. C, Draining lymph node cells from four weeks infected mice were washed 3 times in PBS, spun down onto glass slides and stained with hematoxylin/eosin. Pictures represent the resulting cytospins of infected LN cells showing various forms and degree of infection. Red arrow represented a parasite bound to the extracellular membrane of a non infected cells purified from draining LN of infected mice. (0.80 MB TIF) [file ppat.1000494.s002.tif]

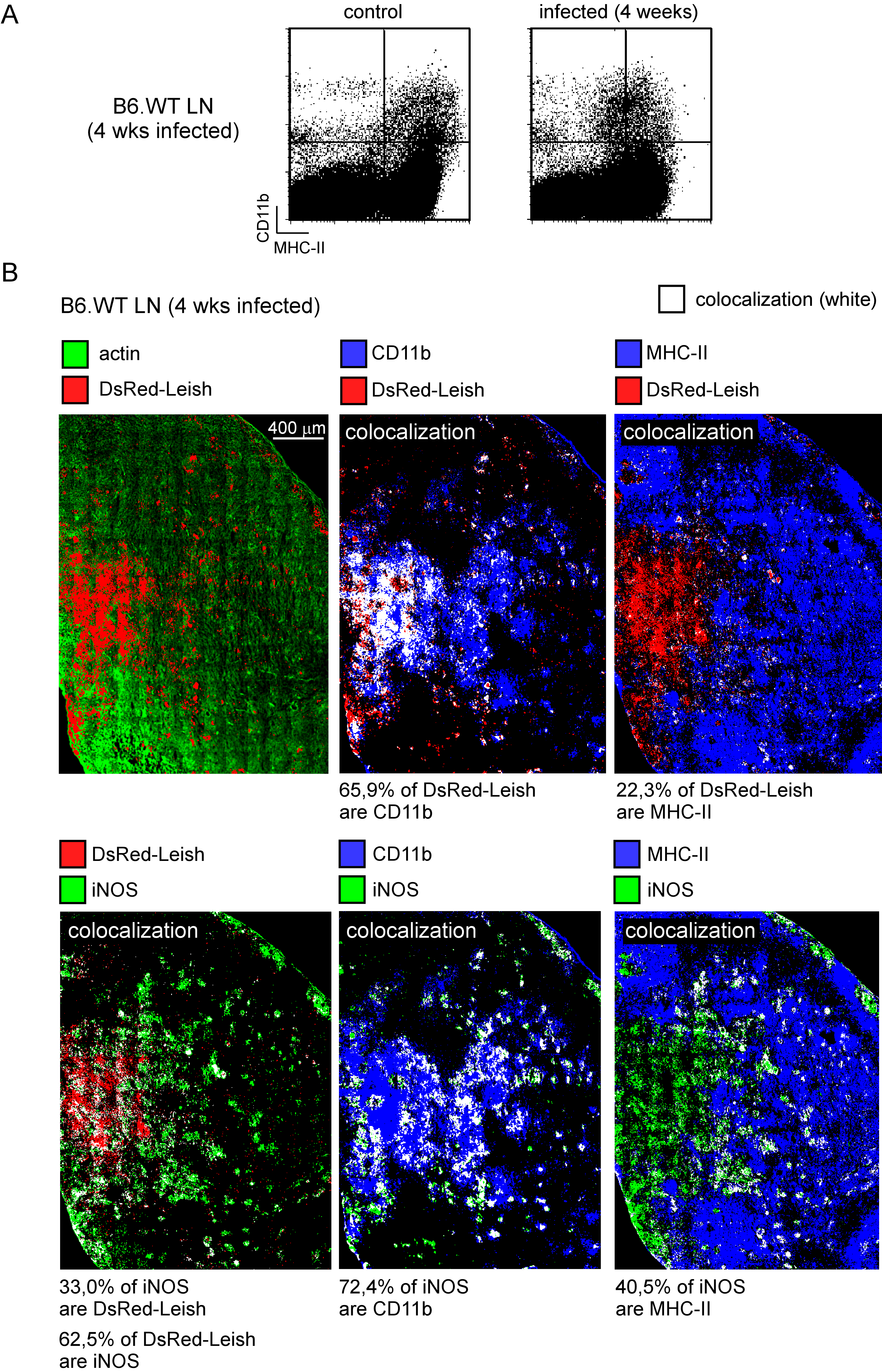

Supplement: Figure S3 — Downregulation of MHC-II expression following L. major infection. Wild-type C57BL/6 mice were injected into the footpad with PBS or DsRed-expressing L. major promastigotes. Mice were sacrificed four weeks post-infection and the draining popliteal lymph nodes were collected. A, Total lymph node cells were analyzed for MHC-II, iNOS and CD11b expression by flow cytometry. B, Serial LN sections were analyzed for CD11b, actin, DsRed and iNOS expression by immunofluorescence. Panels are color-coded with the text for the antigen or fluorescent L. major parasite examined as well as the colocalization. Numbers indicate the percentage of colocalizing cells in the upper panel. Scale bar = 400 µm. Data are representative of 3 independent experiments. (2.66 MB TIF) [file ppat.1000494.s003.tif]

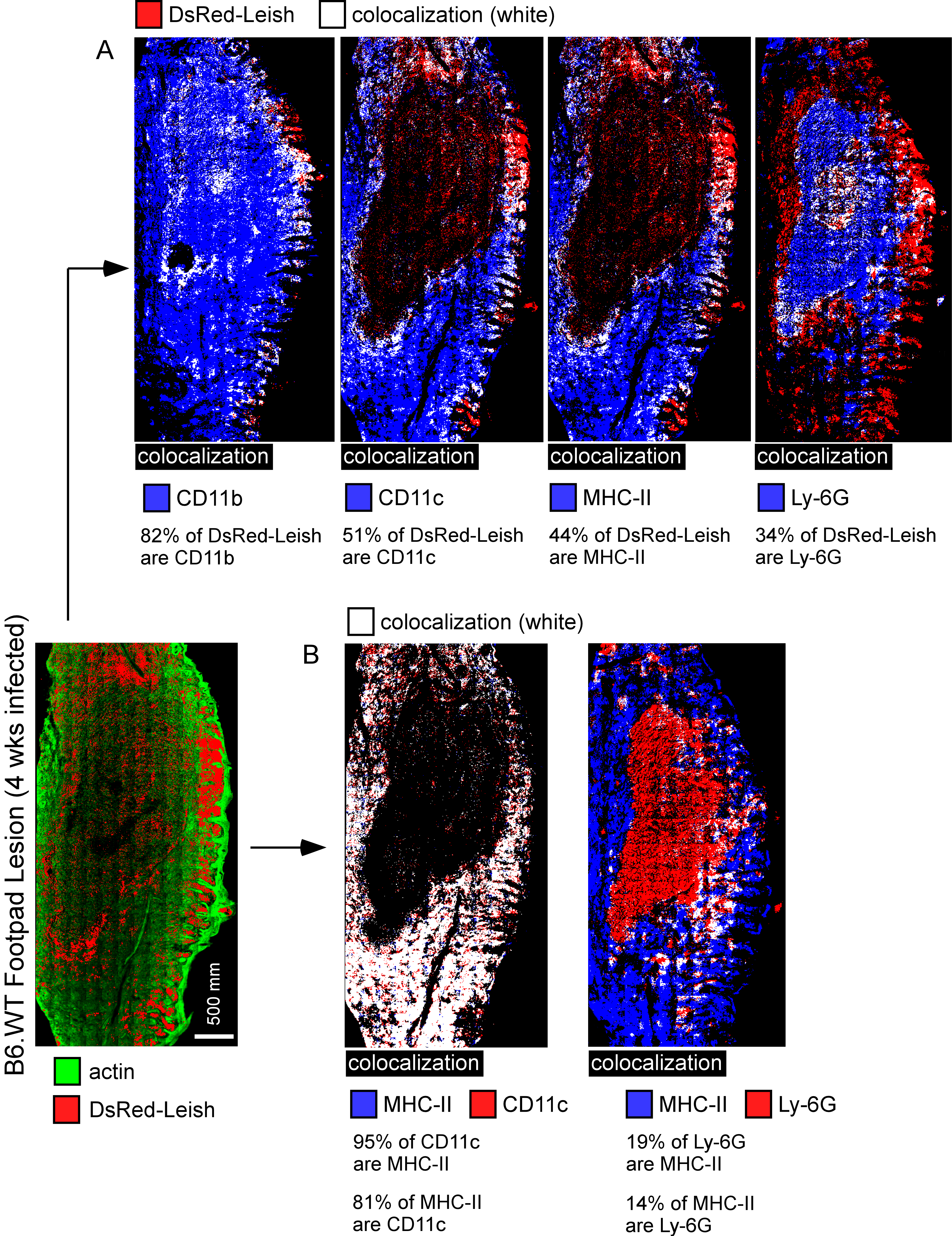

Supplement: Figure S4 — Characterization of infected cells in footpad lesion. C57BL/6 mice were injected into the footpad with PBS or DsRed-expressing L. major amastigotes. Mice were sacrificed four weeks post-infection, footpad were collected and examined by immunohistochemistry. A–B, Serial footpad sections were analyzed for actin, DsRed, CD11b, CD11c, MHC-II and Ly-6G expression. Panels are color-coded within the text for the antigen or fluorescent L. major parasite examined as well as the colocalization. Numbers indicate the percentage of colocalizing cells in the upper panel. Scale bar = 500 µm. Data are representative of 3 independent experiments. (2.24 MB TIF) [file ppat.1000494.s004.tif]

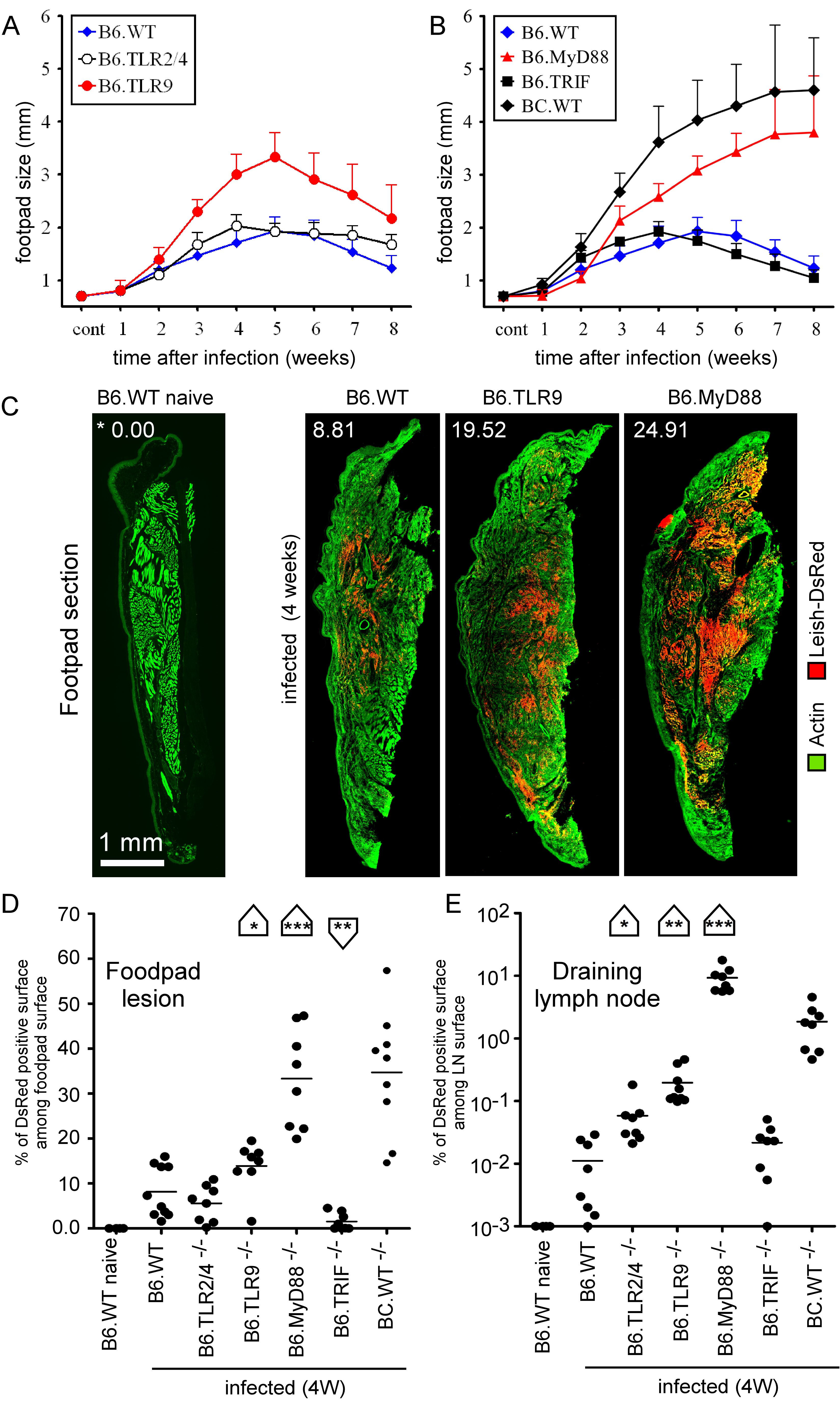

Supplement: Figure S5 — TLR-associated signalling pathways and susceptibility to L. major infection. Wild-type, TLR2/4-, TLR9-, MyD88- and TRIF-deficient C57BL/6 mice as well as wild type BALB/c mice (at least 8 per group) were injected into the footpad with PBS or DsRed-expressing L. major promastigotes (DsRed-Leish). A–B, Size of primary footpad lesions was analyzed during the course of L. major infection for each group of mice. Results illustrate one representative experiment performed with 8 animals of each strain and expressed as means±SD. 3 independent experiments have been performed. C, Naïve and infected wild type C57BL/6 mice as well as infected TLR9 and MyD88-deficient C57BL/6 mice were sacrificed four weeks post-infection, footpad (C–D) and LN (E) were collected and examined by immunofluorescence. Footpad (C–D) and LN (E) sections were analyzed for actin and DsRed expression. C, Panels are color-coded within the text for actin or DsRed-Leish. Numbers indicate the percentage of DsRed-Leish positive surface per footpad surface in the upper panel. D–E, Each data point represents the percentage of DsRed-Leish positive surface among footpad surface obtained from an individual footpad (D) or LN (E) and the data are pooled from two analyses. Student's t test was performed where *, **, and *** denote significance of p<0.05, p<0.01, and p<0.001, respectively, compared to infected wild type C57BL/6 mice. (5.16 MB TIF) [file ppat.1000494.s005.tif]

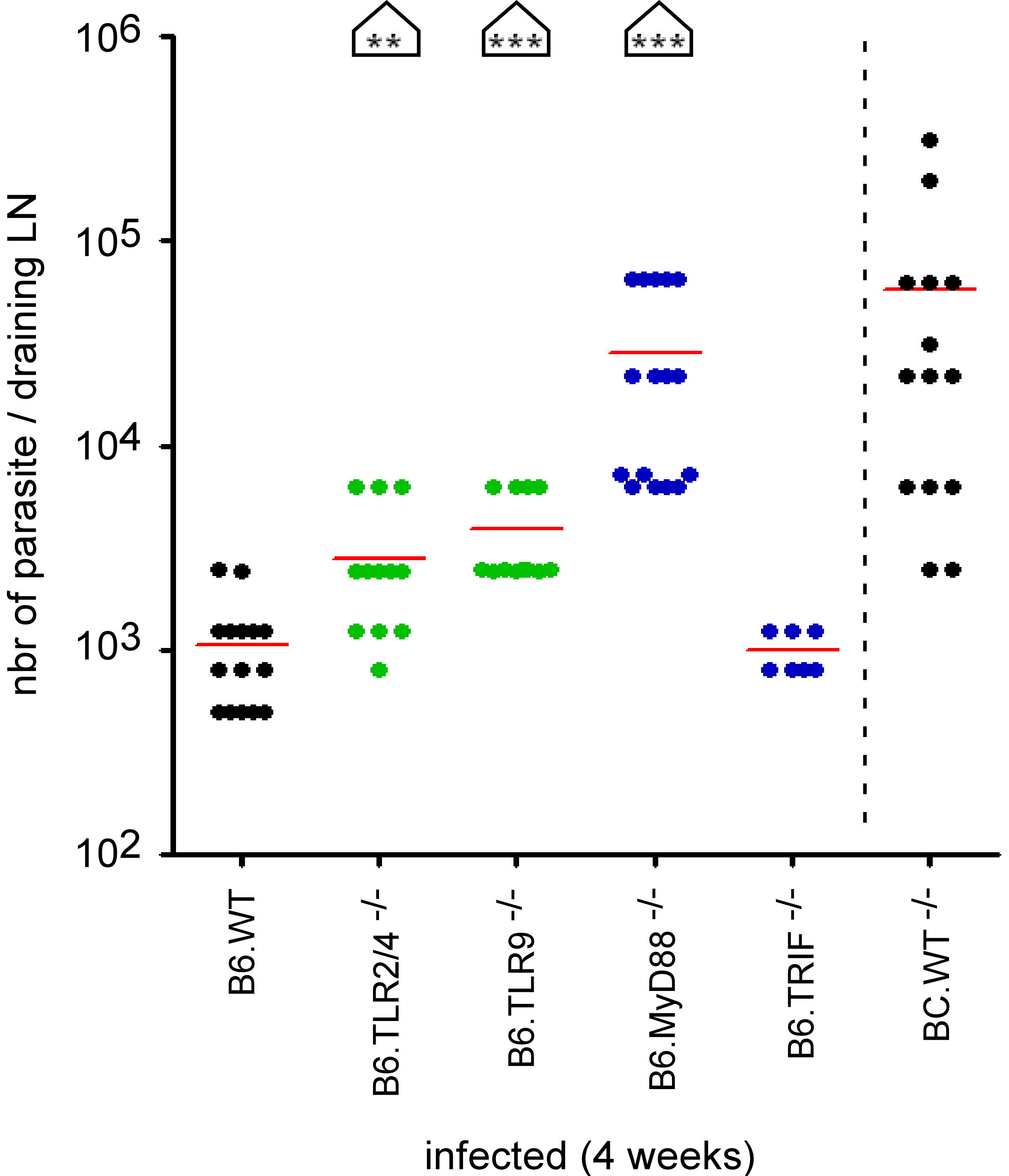

Supplement: Figure S6 — TLR-associated signalling pathways and susceptibility to L. major infection. Wild-type, TLR2/4-, TLR9-, MyD88- and TRIF-deficient C57BL/6 as well as wild type BALB/c mice (at least 4 per group) were injected into the footpad L. major parasites. Each data point represents the number of parasites obtained from an individual LN and the data are pooled from two independent experiments. Student's t test was performed where ** and *** denote significance of p<0.01 and p<0.001, respectively, compared to infected wild type C57BL/6 mice. (0.13 MB TIF) [file ppat.1000494.s006.tif]

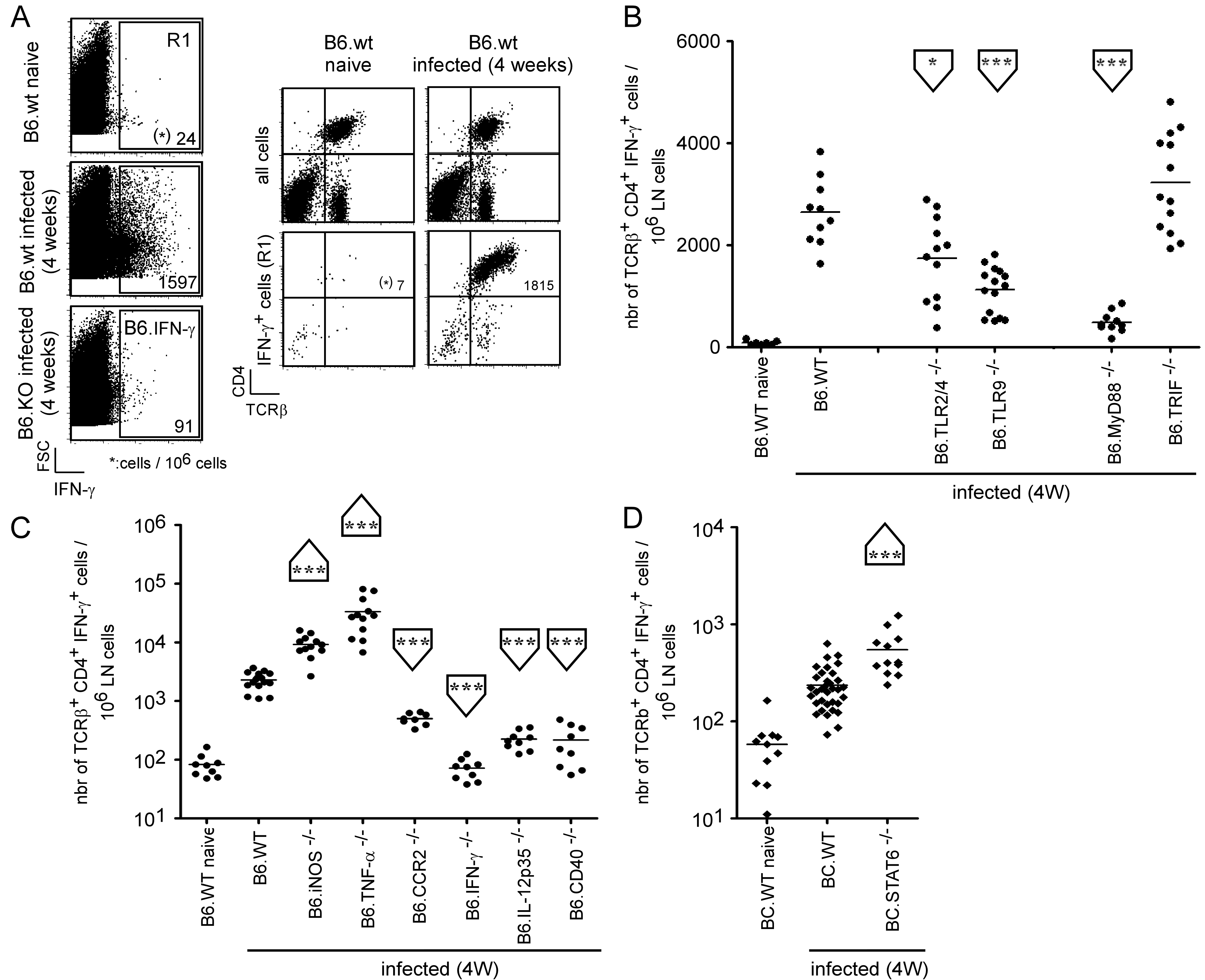

Supplement: Figure S7 — Characterization of IFN-γ-producing cells following Leishmania infection. Wild-type, TLR2/4-, TLR9-, MyD88-, TRIF-, iNOS-, TNF-α-, CCR2-, IFN-γ-, IL-12p35- and CD40-deficient C57BL/6 as well as wild-type and STAT6-deficient BALB/c mice (at least 4 mice per group) were injected into the footpad with PBS or L. major promastigotes parasites. Mice were sacrificed four weeks post-infection and the draining popliteal lymph nodes were collected and analyzed by flow cytometry. A, Total lymph node cells were analyzed for FSC and IFN-γ expression. Total lymph node cells as well as IFN-γ-gated cells (R1) were analyzed for TCR-β and CD4 expression. Numbers in box indicate the number of positive cells per 106 cells acquired total cells. B–D, Each data point represents the number of TCRβ+ CD4+ IFN-γ+ cells per 106 LN cells acquired obtained from an individual LN and the data are pooled from two (B) or three (C–D) independent experiments. Student's t test was performed where *, **, and *** denote significance of p<0.05, p<0.01, and p<0.001, respectively, compared to infected B6.WT (B–C) or BC.WT (D). (0.38 MB TIF) [file ppat.1000494.s007.tif]
